# *Media mastery* and digital devices: sensitizing framework for understanding student experiences of connectivity and control

**DOI:** 10.3389/fpsyg.2026.1742875

**Published:** 2026-03-25

**Authors:** Ingunn Hagen, Ronald E. Rice

**Affiliations:** 1 Department of Psychology, Norwegian University of Science and Technology (NTNU), Trondheim, Norway; 2 Department of Communication, University of California, Santa Barbara (USCB), CA, United States

**Keywords:** connectivity, control, digital devices, *media mastery*, media psychology, paradoxes, university students

## Abstract

**Introduction:**

This article summarizes the concept of media mastery as a sensitizing framework for understanding several paradoxes of digital media use. It then applies the framework to understand ways in which university students attempt to manage their digital devices.

**Materials and methods:**

The paper first provides an overview of the development of the concept. Then, based on focus group data from Norway 2015–2016 and relevant literature reviews, we explore how students attempted to navigate tensions and paradoxes involving connectivity (tensions between relationships and expectations) and control (tensions between dependency and awareness).

**Results:**

Many participants expressed frustration with social media and their own ability to control media use and distractions, questioning whether they should consider themselves dependent or addicted to their devices. They also discussed self-regulation strategies, such as controlling or limiting their digital media consumption, by becoming more aware, in control, and mindful.

**Conclusion:**

For the focus group university students, the benefits of digital convergence (e.g., communication and multifunctionality) were paradoxically accompanied by challenges (e.g., pressure of constant connectivity and digital dependency). The study provides insights into the tensions and paradoxes in the processes of amstering, and being mastered by, digital media.

## Introduction

Over the past few decades, we have witnessed not only a transformation in access to and use of digital media, but also a growing pervasiveness and personalization of these media in everyday life (Germanakos and Belk, 2016; Kennedy, 2008; Yates and Rice, 2020). Young adults, especially, are rarely separated from their digital devices, in particular smartphones, making their interactions, perceptions, and experiences with digital media a crucial area of understanding.

In 2016 there were some main concerns that can be portrayed as gaps in the research literature. There was excitement about the potentials for connectivity, but also concern about the negative sides of constant connectivity with one’s social network, especially through Smartphones which were becoming more sophisticated and available, and the increasing availability of PCs, especially laptop computers that provided easy access to Internet in homes, educational settings and workplaces. Thus, ever more users were able to have constant contact with their friends and families and even extend their network (Hampton, 2016).

However, there were also criticism of and concern about the negative sides of smartphone (and other digital media) use (Cheever et al., 2014). Two issues in particular were increasing anxiety and expectations for constant availability, and users’ Fear of Missing Out (FOMO) when they did not have access to a smartphone, PC or other Internet connected devices for constant connectivity (Przybylski et al., 2013). Table 1 lists some conceptualizations of this general phenomenon. Another concern was about dependency or addiction, especially related to smartphones, with features and apps designed to “hook” young users through habit-forming features and apps (Eyal and Hoover, 2014). In general, there was limited research on how youth were engaging with and interpreting digital media, beyond simple measures of access and usage. Also, there was increasing discussions about how and who should regulate digital media, especially for children: parents, schools, and/or governments. Thus, there was a growing social concern about and research focus on the necessity to regain agency in terms of increased user control, such as through media literacy, awareness, and self-regulation.

**TABLE 1:** Selected concepts related to pressures to be accessible and respond on digital media.

Concept	Representative source
Always on (a pervasive mediated communication environment)	Baron, 2010
Anytime, anyplace connectivity (removing obstacles of time and place for all communication participants)	Vanden Abeele et al., 2018
Autonomy paradox (employees’ mobile phone use increasing flexibility and control while also generating collection availability expectations and reduced autonomy)	Mazmanian et al., 2013
Availability norms	Geber et al., 2024
Connected presence (an interconnected blending of both copresent and mediated interactions)	Licoppe, 2004
Connective availability (dyadic partners perceive each other as continuously available, representing security and protection, and subsequent better well-being)	Miller-Ott et al., 2014; Taylor and Bazarova, 2021
Mobile entrapment (anxiety, guilt, stress to be available via mobile phone)	Hall, 2017
Mobile maintenance expectations (others’ presumptions that a user will maintain their mobile availability)	Hall and Baym, 2012
Permanently on call (social pressure to be available)	Halfmann and Rieger, 2019
Perpetual contact (from limited, effortful, delayed communication to a pervasive media environment)	Katz and Aakhus, 2002; Rule, 2002
Soft coercion (reciprocal expectations of availability)	Ling, 2016

Little of the vast research on these issues as of 2016 included two of the present paper’s foci: First, the simultaneous inherent tensions and paradoxes between the positive and negative aspects of digital media, and second, an overall framework that provides a lens to illuminate those tensions. To help understand these phenomena, we apply the media mastery framework, which highlights the dual nature of media use: users attempt to master their use of media, but they are also influenced or even mastered by the same media (Rice et al., 2018). While *media mastery* implies focusing on people’s responses, perceptions, and experiences, the framework also emphasizes the role of digital media in influencing or generating the premises for those perceptions, feelings, and behaviors. Thus, the core interest is about tensions and paradoxes between both forms of *media mastery*, and how to manage them.

The media mastery framework is inspired by the field of media psychology, which focuses on the relationship between human behavior, psychological processes, the digital media that surround us, and their use and effects. According to Dill, “Media psychology is the scientific study of human behavior, thoughts, and feelings experienced in the context of media use and creation” Dill (2013, p. 6). Rutledge (2013, p. 43) argues that its focus is on the human and relationships among media users, creators, and distributors, and not the media technologies *per se*: “media psychology seeks to understand the intersection of human behavior and technology to connect the positive capabilities of technology with human needs and goals so that individuals and society can grow and flourish” (p. 43).

We first explain the concept of *media mastery* as a framework for (including a typology of) components critical to understanding issues of digital *media mastery*. We provide examples from prior studies of its use as a sensitizing analytical framework. Then we illuminate how the media mastery framework is a useful starting point for examining two of its components. First *connectivity aspects*: how students discuss positive and negative aspects of connectivity, and where the balance of different domains lies in their digital media use. Second, *control aspects*: whether students report a more or less a sense of dependency or even addiction, and of awareness and self-regulation within the context of their digitally connected lives. To explicate our position, the following section explains the media mastery framework.

## Background—What is the *media mastery* framework?

### Brief summary of the development of the framework

The concept of *media mastery* has been introduced and developed in two articles (Rice et al., 2018, 2020). The idea emerged around 2005 from noticing students’ concerns about attempting to manage multiple media when they began to have access to both mobile phones and desktop and laptop computers as frequent digital devices. The 2018 article presented an initial version of the media mastery framework. Based on insights from focus group interviews conducted in Norway and the U.S. in 2005–2006 and in 2015–2016, as well as additional literature synthesis relevant to students’ digital media practices, the article extended that discussion by further developing the framework, and using that to categorize and examine relevant studies in the field, and to frame analyses of research data.

**FIGURE 1:**
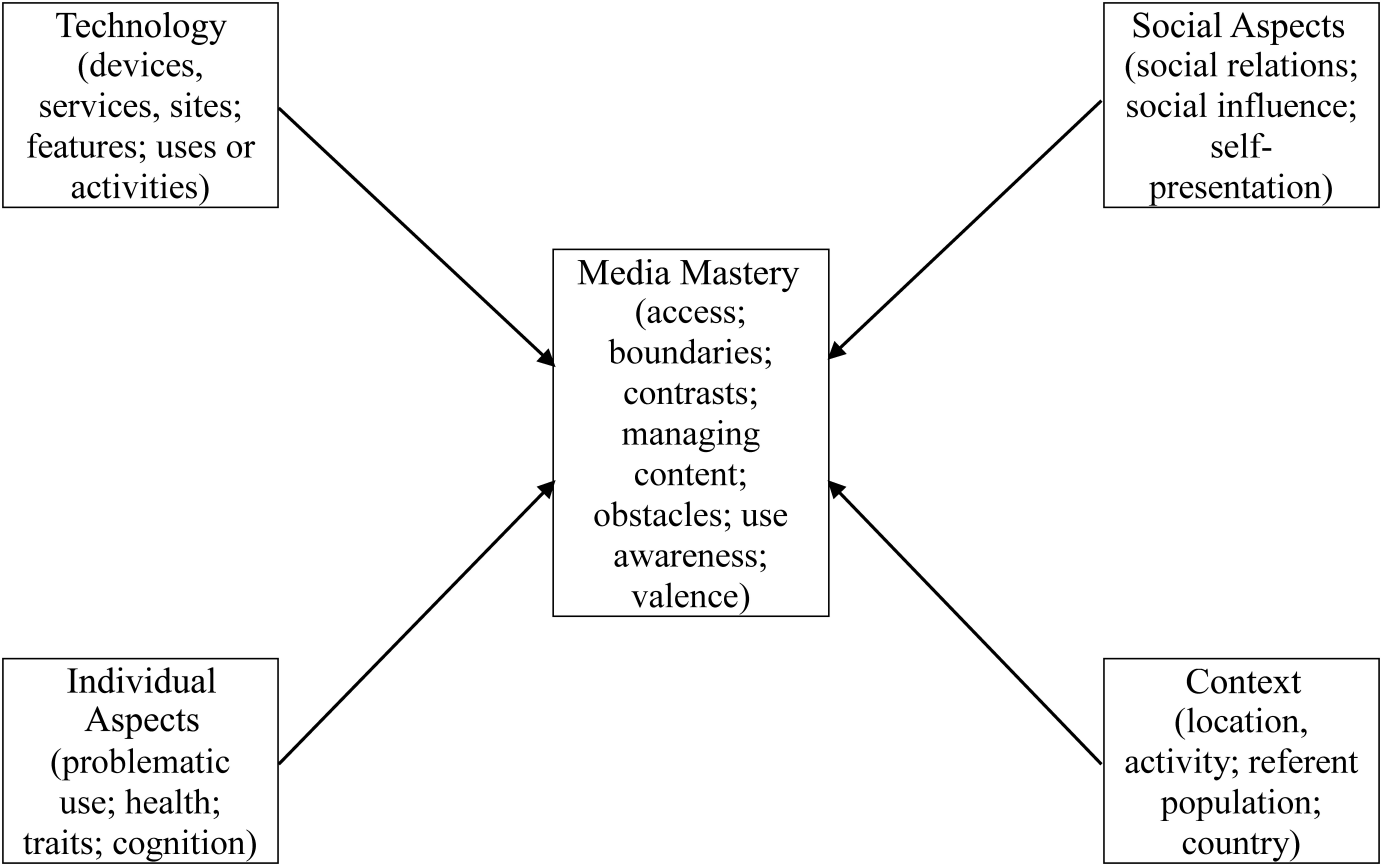
Simplified media mastery framework: main components and subcomponents. The four corner components, which provide occasions for *media mastery*, are also interrelated, and over time *media mastery* experiences can influence each of the occasion components.

The revised framework includes four components that provide occasions for media mastery, each with major subcomponents—*Technology* (the technologies, platforms, and uses); *Social Aspects* (the social and relational aspects and contexts); *Individual Aspects* (those involved in or arising from or associated with use); and *Context* (location, activity; referent population; country)—and the media mastery component itself (aspects related to use, management, awareness, and implications of technology). Figure 1 shows the main framework components and their subcomponents (see Rice et al., 2020, Tables 9.1 and 9.2 for details, including sub-subcomponents, for a total of 140 entries in the media mastery framework). While all five of the main components are interrelated, we here only focus on the media mastery component. This framework provides a comprehensive typology for use in content analysis of literature reviews and in research studies (for such a review, see Rice et al., 2020).

Based on these analyses and revisions, we redefined *media mastery* as “the more or less conscious and more or less successful ongoing process of how people understand, manage, make sense of, cope with, and use one or more new media in their everyday lives, as well as how media in turn come to manage, control, or affect individuals and their social relations …. how we master the balance and use of one or more media in different contexts” vs. “the ways in and extent to which these media master us—as our activities, concerns, and relationships are being shaped through, facilitated and constrained by, and dependent upon, the use of these media” (Rice et al., 2020, p. 3). This includes not only how people use the media, but also the choices they make, the habits they form, and the social interactions shaped by their media use. Emotional wellbeing plays a key role as well: for example, do platforms like Facebook or Instagram make users feel competent, connected, and included—or do they provoke feelings of regret after unintended time spent scrolling—or both? *Media mastery* extends beyond usage—it reflects how digital media use may shape our thoughts, emotions, concerns, actions, and relationships, and how we may apply forms of control, awareness, and self-regulation to shape the choices, uses, and effects of digital media.

Four key assumptions underpin the media mastery framework: (1) There are reciprocal relationships among the five components; (2) Users more or less successfully actively attempt to understand, interpret, and manage both the positive and negative consequences of media use; (3) Media experiences are inherently filled with paradoxes, tensions, and contradictions; and (4) *Media mastery* is highly contextual, shaped by individual and social values, attitudes toward media, user motivations, and the features and affordances of specific media.

While the *media mastery* concept overlaps with *media literacy* (see O’Neill and Hagen, 2009; Potter, 2022; as well as other media theories—see Rice et al., 2020, pp. 253–254), it is more process-oriented and more comprehensive. It draws attention to the role of media in shaping daily habits, involving ongoing self-regulation, negotiation, and tensions. Unlike digital media literacy, which emphasizes awareness and knowledge needed to help users better *master their media use*, *media mastery* also considers how people may be more or less *mastered by the media they use*. As argued by Rice et al. (2020), “the media mastery framework allows a more analytical approach, by integrating a variety of prior and new perspectives, highlighting the relevance of many diverse concepts, and revealing associations among many otherwise disjointed or typically unlinked concepts” (p. 42).

### *Media mastery* as a sensitizing analytical framework

We have used *media mastery* as a sensitizing analytical framework in our research on students’ digital media use. Faulkner (2009) describes sensitizing concepts as central organizing ideas. The framework helped us explore how engaging with one or more forms of digital media requires navigating paradoxical dynamics in usage and outcomes (Rice et al., 2018, p. 1). For example, one of our central findings—based on both focus group analysis and literature reviews—was the ongoing tensions and paradoxes between the benefits and drawbacks of using separate or multiple forms of digital media. Innovations in general, such as new media, tend to invoke both positive and negative consequences, direct or indirect, intended or unintentional, short-term or long-term (Rogers, 2003). de Sola Pool (1983), a pioneer in media research, described traditional and newer media as having “dual lives,” offering simultaneous benefits and drawbacks. We used the term *paradox* to reflect how such opposing implications often coexist in digital media use (Rice et al., 2018). These effects frequently lack clear resolution and may operate simultaneously. Users may or may not be aware of these implications, may or may not attempt to resolve them, or may or may not be able to do so. In an earlier literature review, we outlined positive, negative, and paradoxical aspects of how adolescents and college students used mobile phones and PCs (Rice and Hagen, 2010). Through focus groups and a later literature review, we identified five paradoxes central to *media mastery*: stimulating yet exhausting, frustrating yet beneficial, structured yet chaotic, flexible yet uncontrollable, and an abundance of content versus content of value (Rice et al., 2018, p. 17). As digital media continues to evolve in form and complexity, these paradoxes may persist, and new ones may arise.

Thus the present article applies *media mastery* as a sensitizing analytical framework to identify and describe how users manage multiple media across physical environments such as home, school, and work, and within various social contexts, and to refer to “the ways in and extent to which these media master us—shaping, facilitating, constraining, or defining our activities, concerns, and relationships” (Rice et al., 2018, p. 3).

### Related *media mastery* research: connectivity and control, balance, or dependency?

This section summarizes three other studies applying the Media Mastery approach to focus groups of the overall project. Two MA students affiliated with the first author’s university wrote their MA theses based on the six 2015–2016 Norwegian focus groups.^1^ The third was conducted by a graduate student and members of the research group at the second author’s university, based on the six 2006 and six 2016 U.S. focus groups. Here, we note applications and implications of media mastery components related to the main themes of our study, tensions and paradoxes between connectivity and control, balance and dependency, as well as how the media mastery framework can intersect with relevant media theories (i.e., work-life border theory, media domestication theory, and privacy calculus).

### Balance, awareness, and addiction

Aanby (2017) examined the role of technology in disrupting students’ balance between life and work. Her thesis drew on work-life border theory and work-life literature to explore how students attempted to maintain a balance between “work time” and “leisure time,” two central domains (Clark, 2000, p. 753). Many of the interviewed students identified social media as a key source of life-work interference, as digital media use often blurred the boundaries between work, school, and private life. Further, Aanby (2017) described how several students perceived themselves as addicted to or dependent on digital media, while others were aware of or concerned about this possibility. Thus, the media mastery framework helped identify students’ experiences of tensions among balance, awareness, and addiction in their use of digital media.

### Control and connectivity

Fløtra’s thesis (2019) invoked domestication theory—how users “tame” and integrate media technologies in their daily lives (Silverstone et al., 1992)—and *media mastery* as the conceptual frameworks. Fløtra interpreted *media mastery* as the ability to understand, relate to, and incorporate media into daily routines, central to media domestication. She emphasized that successful (good) domestication was a form of balanced *media mastery*. Her thesis addressed how students managed digital media use across different settings—home, study, work—and whether they felt they were in control of (mastering) or controlled by (being mastered) the media. Dependency emerged as a key theme. The interviewed students described this dependency as emotionally complex and connected to social bonds, not solely negative. Several of the interviewed students admitted to experiencing fear of missing out (FOMO; Przybylski et al., 2013) as a significant factor in their perceived need to remain constantly available. Her core conclusion was that students were “masters without control”—technologically knowledgeable but lacking in self-regulation. She suggested that the students’ moral judgments and actual practices were misaligned, generating frustration and perceived dependency. This study also highlighted tensions and paradoxes of the simultaneous utility of, but dependency on, digital media.

### Controlling privacy boundaries

Wang et al. (2025) analyzed the 2006 and the 2016 US focus group discussions to assess continuation of, or change in, themes about online privacy boundary issues in the students’ use of multiple digital media. One example of the emphasis by the media mastery framework on tensions and paradoxes is reflected in the “privacy calculus model” (Dienlin and Metzger, 2016), whereby users may more or less consciously or knowledgeably trade off perceived benefits from digital media use (mastering use) against perceived privacy and surveillance risks (being mastered). Grounding their research in that perspective, Wang et al. (2025) applied 13 of the 22 sub-subcomponents of the boundaries subcomponent of the media mastery component (anonymity, audience, context collapse, parental access, privacy management, public, safety, self-broadcasting, surveillance, trust, visibility-transparency, vulnerability, and watchfulness) to the focus group transcripts via reliable content coding. Attending to these components in the focus groups helped identify direct and indirect manifestations of privacy issues in the discussions, compared over the decade between focus groups. The most frequently mentioned or implied concerns across both periods included audience, visibility-transparency, self-broadcasting, and privacy. This study shows how the media mastery framework can help identify and apply a range of specific concepts from the broad digital media use and effects literature.

## Two digital *media mastery* concepts: connectivity and control

Aligned with recent research on digital media and social media usage (e.g., Pugno, 2025; Rasmussen et al., 2020; Rice et al., 2020; Syvertsen, 2020; Twenge, 2019), and related to the media mastery framework and research, we apply the framework to investigate how university students experienced two main subcomponents of the media mastery framework. These include *connectivity* (related to sub-subcomponents such as social relations, social influence, and self-presentation; and availability expectations, perpetual contact, boundaries), and *control* (related to sub-subcomponents of addiction, dependency, habit, or problematic use; and use awareness, including balancing kinds of use, choices, expertise, filtering, self-regulation, strategizing, and multiple media). We then relate our results to relevant research on these topics.

RQ1: *Connectivity (relationships and expectations)*. How do students engage with and manage multiple digital media throughout their day—waking up to smartphone alarms, navigating various apps for everyday tasks, texting and phoning friends and co-workers, and transitioning to computers for academic work—while also contending with social disruptions and connectivity expectations from peers, family, and friends?

RQ2: *Control (dependency and awareness)*. Moreover, to what extent do students feel aware of and in control of their digital media habits? Do they perceive themselves as dependent on—or even addicted to—these technologies? When students view their digital and social media use as problematic, are they able to control or regulate it? And if not, does this lack of control impair their sense of balance and wellbeing?

## Method: summary of Norwegian digital context, and focus group process and samples

### Norwegian digital device context

Norway at the time was one of the most digitally advanced societies in the world, with almost universal internet access and daily use. Indeed, in 2015 97% of the Norwegian population over 12 years had internet access at home, at school or work, or elsewhere, and 90 percent were using the internet daily (Ministry of Digitalization and Public Governance, 2016). The 2016 Norwegian Media Barometer reported a clear increase in time spent on the internet, with young adults being the most active users (Statistics Norway, 2017). Internet use was positively associated with more education, and male gender. Smartphones had in 2015/2016 overtaken PCs role as the primary digital device among young adults. Based on a nationally representative survey of 2,888 children and young adults (Norwegian Media Authority, 2016), even by 2016, 97% of Norwegian children between 9 and 16 had access to a mobile phone. They were more likely to obtain news online (37%) than from TV (27%) or printed papers (9%). A total of 80% used search engines weekly, increasing with age. Tablets were also widely owned, but not so central to younger people. By 2015–2016, smartphones were the dominant personal device among youth and young adults in Norway, used heavily for communication, social media, entertainment, and news. Still, PCs were widely used, especially for school, work, and some entertainment. However, PC use was declining relative to smartphones (AudienceProject, 2016; Statistics Norway, 2017). By age 16, 85% used social media at least daily, with use increasing with age. In 2015 Facebook was the dominant social media platform. This usage was gradually diversifying and the most popular platforms among young adults became Facebook, Instagram, Snapchat, and YouTube (see AudienceProject, 2016). Young adults (18–30 years) were pioneers in the shift from traditional to digital media use, and especially mobile-first media consumption.

### Sample: focus groups

We conducted six focus group interviews in Norway during 2015/2016.^1^ To control for possible gender differences in focus group discussions (see Hollander, 2004), we wanted to conduct two groups with all females, two with all males, and two mixed. However, due to recruitment challenges, we ended up with two female groups (*N* = 3,4), one male group (*N* = 3), and three mixed groups (N F,M = 3.1; 1.4; 2.1) for a total of 22. The participants were recruited from the populations of Bachelor students in Psychology and Media Studies. The recruitment age was defined as 18–30 years, but the majority were in their early to mid-twenties. Most of them were Norwegian, from the region of the main campus of the university, but also from other parts of the country.

Each group was asked the same seven questions, first for personal computers, laptops, and tablets, and second for mobile phones. These included questions about use purposes, school-related purposes, personal or social purposes, achievement of goals through the device, negative experiences, situations where using the device was enjoyable, and what life would be like without the device. Five of the focus group interviews were in Norwegian, one in English. The discussions were recorded, and transcribed into digital files, and the five Norwegian files were then translated into English.

### Thematic analysis of focus group discussions

We combined all answers from all focus groups about both sets of media from the transcripts and conducted emergent and reflective thematic analysis of the discussions, using NVivo11. Thematic analysis is a widely used, flexible, and efficient way of identifying themes in research data (Braun and Clarke, 2006; Braun et al., 2022). We followed these analytic steps: (1) Getting to know the data, though reading and rereading, where we also noted ideas; (2) Initial coding, guided by relevant research literature and MM concepts, identifying meaningful codes in an inductive manner; (3) Searching for themes, where we grouped different codes into potential themes; (4) Reviewing the themes, which implied refining, reconsidering, and reorganizing themes; (5) Defining and labeling the themes; and (6) Producing the report, where the identified themes were discussed, and related to the relevant research literature and research questions. The media mastery framework is not a theoretical causal model, so the qualitative analyses and results were not hypothesis tests.

### Positionality

The first author is Professor of Psychology (MA Clinical Psychology, MA Communication, Ph.D. Media studies), focusing on media and communication psychology, at a Norwegian university, primarily from an interpretive qualitative perspective. The second author is Professor of Communication (MA and Ph.D. Communication Research), focusing on organizational communication and media, at a U.S. university, primarily from a post-positivist quantitative approach. They have collaborated over many years, integrating their perspectives through paradigmatic critiques and suggestions. Thus, the larger *media mastery* project involves a range of methodologies, including literature reviews, focus group interviews, *a priori* and emergent and reflective qualitative analysis, and quantitative analysis, along with theoretical foundations for the components and overall typology. They iteratively discussed and refined the themes, referring to the overall typology, while jointly questioning and revising their contributions. The process and overall framework were grounded through their past publications. The integrated and iterative dual paradigm and mixed-method collaboration between the two authors have informed our decisions, analyses, and writing.

## Results: social connectivity and control (digital dependency and awareness)

As in qualitative research generally, the analysis already began in the focus group interview situation, where the researchers (moderator and assistant) were present, asking questions and facilitating the discussion. The assistant took notes about main points in each response while the entire focus group discussion was also digitally recorded and then transcribed. The interview guide and notes were used to help identify possible initial themes, and the transcripts were used to fill out and contextualize the themes.

Then a thematic analysis approach was used, as described above. We applied reflective thematic analysis, which has been practiced and discussed in recent years (see Braun and Clarke, 2006, 2021; Braun et al., 2022). Thematic analysis is about identifying “emerging themes”; patterns based on the initial and meaningful codes, through an inductive process. Here, however, we also located themes within components of the sensitizing analytical framework of *media mastery* and on relevant research literature and were conscious of and reflective about our own perceptions of university students’ use of digital devices.

Five main themes emerged: *Device Convergence*, *Context-Dependent Technology Use, Social Connectivity*, *Digital Addiction/Dependency*, and *Digital Awareness*. The five themes captured essential aspects of how participants navigated and mastered digital communication media. The first two reflected *contextual* components, the third the main concept of *connectivity*, and the latter two the main concept of *control*. The first two themes were not conceptualized in the two RQs, but emerged as background contexts/precondition for some of the participants’ experiences of social connectivity, digital addiction/dependency, and digital awareness.

### Context precondition: device convergence

Essentially, digitization of formerly analog data, digital transmission networks, and software-based digital devices have blurred distinctions among media, leading to digital convergence (Jenkins, 2006; Dal Zotto and Lugmayr, 2016). Discrete traditional media could be considered as artifacts of analog data and medium-dependent material formatting (Rice, 1999). However, once digitized, the same content could be conveyed to and displayed through a wide variety of devices, media, or platforms, often in modified form, and sometimes simultaneously. Further, each device could offer both distinct and similar features, taking advantage of the plasticity of digital software and technology. See also Rice et al. (2023) for an analysis of how changing and converging forms of personal computers and mobile devices affected use of online activities during the second decade of this century. Thus, due to such technological developments at the time of the focus group discussions, the students’ digital devices possessed increasingly multiple and overlapping functions, or device convergence.

The participants seemed to be concerned with these changes—as digital convergence meant that a device could partially substitute for another when needed, but also that there were more and more features and capabilities to learn and use. This convergence was particularly significant for the mobile phone as it evolved into the smartphone, which could substitute for other devices, like PC, MP3 player, camera, alarm clock, physiological measurement tools, news source, watch, etc. Participants discussed how technological advancements led to such multifunctional devices, eliminating the need for multiple separate devices. For example, participants reflected on the convenience of carrying fewer devices while still accessing necessary functions:

*1: I think that the mobile phone has taken over much of the function, like compared to before when we had Mp3 players and all that. Yes, a lot was separated then, and now it has all in way just been converted into one thing. Thus, you do not have to walk around with a mobile phone and Mp3 and all sorts of strange things.*

*2: I managed well without my laptop for a while. (*…*) I had my tablet, it’s kind of a mixture, but it has a smartphone platform, right, just with a larger screen.*

*1: But now you can get these hybrids, they are a mixture of computer and tablet. That leads to an unclear dividing line.*

The participants shared examples of different instances where the mobile phone substituted for other devices.

*1: It’s perhaps, primarily I use it as an alarm clock to get up in the mornings. I use it as a watch; I don’t wear a watch anymore. It’s the phone like, I don’t need a watch anymore, don’t need an alarm clock. (*…*) just taking pictures and filming for instance, because no one brings along, or some people do bring their camera, but it’s not like before. Thus, the mobile has taken over that, so that is somewhat a joyful thing, looking back at things you’ve done.*

*2: And I feel that PC functions in a way can be allocated on to other things as well, so the void is somehow not so big then, I feel.*

*1: Yes. Mobile can do both. If you have additional functions.*

*3: Exactly.*

Thus, participants discussed device convergence—the distribution of digital content across many devices, and the merging of multiple digital functions into fewer devices—as a precondition for modern digital media, and shared instances of how they experienced this.

### Context precondition: appropriate device use

The students were asked about computers and mobile phones separately in our focus group interviews, and thus probably (and intentionally) increased the distinction between the use of these devices. However, the participants also highlighted that technology use was highly dependent on context. While the interviewed students stated that computers continued to be important to them (along with, at that time, the relatively newer, additional use of laptop and tablet computers), they reported that their use was more context-specific compared to smartphones. Students reported that their smartphones accompanied them across a broader range of situations and locations and were the interface to their social networks. The participants indicated that computers were primarily used for work and school tasks, while mobile phones were associated with and used more for personal and social interactions. As described by one interviewee:

*Actually, I use my phone much more than my Mac. The Mac is almost only used in work related to school. This is because it’s much easier to check Facebook on my phone.*

Furthermore, there seemed to be a variation in where and when people used their mobile phone and computer, often based on where they were:

*It depends on the day, where you are and stuff like that. When I’m on the bus then I use the mobile. I do not use a laptop when I sit on the bus, as that is too much. But if I am sitting at home then I use the mobile less and the PC a lot more.*

Mobile phones were portrayed as favored for on-the-go activities and social and personal interactions due to their convenience and immediacy, while computers were said to be preferred for tasks requiring reliability and thoroughness, such as checking school-related information. The participants said that they trusted computers for accuracy, especially when handling important matters like academic websites, preferring to double-check information on a PC rather than a mobile phone. In the words or one participant:

*But I notice stuff like Student web and such school-related stuff I usually use my PC to, really double check. I don’t know, but I have such a fear that the mobile phone does not show everything correctly, so I have to double check everything on my PC. But I use my mobile mostly for non-school related items.*

This contrast suggests that participants assessed their devices based on context-specific aspects such as portability, trust, functionality, and timeliness.

### Connectivity and the “availability norm”

The concept of *social connectivity* effectively captured the recurring theme of digital connection discussed in the focus group interviews. Participants described various ways they used their computers or mobile phones to nurture social relationships.

*1: I like talking on the phone, with family or friends that are not with me but somewhere else.*

*2: For me as well. I enjoy talking to some of my very best friends, good friends, they are living in different places, Japan or Canada. I can use my*… *What do I say, there is a difference between the mobile phone and a smartphone. So, this smartphone can help me to have conversations with them. The internet is really cheap, it is actually free, so I can share my feelings with my friends. But if it was not there, I could not.”*

Another participant described: *“When I am Skyping with my kid - that I really enjoy. When I can see my parents through computer or skype or whatever it is.”*

Beyond maintaining contact with family and friends, the participants reported that digital media also played a role in initiating and sustaining romantic relationships:

*1: I think the biggest joy I’ve had with mobile usage really is my ex, who I found through using my mobile actually.*

*2: I achieved my love using mobile phone. Yes, it is true because I used to talk a lot, hours after hours after I met my wife, when I was kind of 19 and she was kind of 16 or 17 and I was 18, 19. Yes, I took her number and then I had to, yes, a lot of conversations. But yes, the mobile phone. Maybe in the past, they used letters, for me it was the mobile phone. Sometimes Facebook also worked, but on Facebook I cannot talk, so yes mobile phone helped me achieve my love.*

Yet, this constant connectivity also brought challenges. Some participants expressed frustration about always being online:

*When everything goes back-to-back, it melts together: computer time, mobile and somehow, you constantly have someone or something to talk to, and so you won’t get time alone to think like. Everything is just messy from when you wake up in the morning and until you go to bed at night. So, you are constantly talking to someone. It’s about everything and nothing. S*… *If people had some time to break up the pattern, relax for a while and like stop for a second and be bored, think a little, it would have been interesting for many, I think.*

The constant accessibility of digital media, especially social media, was said to generate the expectations by others to always be available, an emerging “availability norm”:

*And that you always have to be available. You must bring it everywhere you go, or you feel like you are going to check your phone and then it’s like “Oh no. I didn’t bring it.”*

*And also, there is this thing that you always have to be, or the expectation that you must be available if you have a mobile and a PC, so there’s something about that. It’s like if you [have] access to them both why aren’t you available to get hold of?*

When asked about privacy, participants revealed the tension between availability and personal boundaries:

*1: Yes, privacy is nice but, you’re available all the time, people can reach you. You can choose not to answer, but as he said, there is this expectation about your answering.*

*2: Yes, it’s like this pressure, it’s a demand or*… *But you can hide pretty well if you want to, I think.*

*3: I don’t think so.*

*2: You don’t?*

*3: No, it’s impossible nowadays, unless you log off everything.*

### Control: digital addiction or dependency

Various problematic aspects of digital media use emerged as key themes. A recurring concern among participants was the experience of digital dependency, or what some framed as an addiction to their devices. One participant described symptoms of dependency and withdrawal:

*1: And last time, if you remember, I forgot my phone at home, and then I felt I was missing something. (Laughing). I missed it, and I went back and brought it, because I felt like, if somebody is calling, how am I going to*… *I could not even stay one day without it, so I went back and brought it.*

*2: You become addicted.*

*1: Yes, that is how I knew.*

One focus group discussed whether their behavior should be considered a habit or an addiction:

*1: Yes, I think of habits in the sense that you open the fridge every 15 min to check what’s in the fridge, just for the heck of it. But when it’s gone, there is a break in your routines. It characterizes a big part of its use that and it is something you are accustomed to do, and then it’s taken away from you and it’s like crap. There is something that is missing, but you adjust to it gradually. But it is as he said, it is addictive in the sense that it has some important functions, in certain contexts.*

*2: But I think that most people are quite addicted to their mobile phone. Cause if you notice that you have received a message, it starts to tingle in your fingers urging you to check.*

*3: YES, and becomes like a withdrawal then if you don’t, right. So, in that way you can look at it like an addiction, however I understand what you mean by the habit thing also, because it’s like*… *it is a habit to check here and there*… *If don’t have it like during the week we had, then it’s like. I mean it is all okay, but you feel like there is something that, it is stuck in your head, that you need to check something, right?*

*1: Yes, I take that meaning a little bit literally, being addicted to cocaine, that is a physical dependency that you are physically affected. But I feel like this has a little more to do with habits, but it is as he said, it’s context based as well.*

Participants also expressed dependency on both mobile phones and computers. One person remarked on the phone: “*In any case, it is like glued to my hand.*” Another spoke about computer use*: “Sometimes when I get addicted, I also use the laptop at night—it’s really bad.”*

Group members generally agreed that this digital dependency might stem from a need to feel connected or to avoid missing out. As one put it:

*1: Ehm it’s worse when I’m at a friend’s place the whole evening and I don’t have their Wi-Fi password, because I’ll be sitting there for several hours without internet. I then get this “oh crap” something must have happened feeling, so I need to check it out.*

*Interviewer: Yes, you get kind of restless?*

*1: Yes, I do. I don’t know, I feel like I’m missing out on a lot.*

Other participants shared similar stories:

*3: It was the weirdest feeling when the battery on my phone died on Sunday. It was still early, but I had forgotten to charge my phone during the night. And so, you’ll just walk around feeling completely lost. It’s like you’re not included in the world anymore.*

*2: You feel very naked, yes. You get a little bit of panic.*

*3: And very restless and uncomfortable, kind of. Yes.*

*3: It’s a weird feeling, you like don’t feel like you are with others anymore, you feel left out.*

*2: Scared of missing out on things.*

### Control: digital awareness

Despite the often-overwhelming nature of digital media, some of our participants reported a different perspective. These students told how they consciously resisted following what they perceived as the prevailing crowd behavior regarding digital media use. One shared:

*I think it is quite relaxing because I purposely choose to have a small mobile data because, I would rather not be sitting on my mobile phone the whole time, so I only have internet access when there is Wi-Fi in the area. I’ve heard the terms FOMO and JOMO, FOMO is “fear of missing out” and JOMO is “joy of missing out,” I’m a little more into JOMO, I think it’s a little relaxing to miss out, so I don’t have to deal with stuff. Like every time I would take the train from Oslo to Kongsberg, this person started talking to me like, yeah, I noticed that you’re not on your mobile like every other person (laughs). He thought it was a bit special, so I said I like to observe nature and experience the train ride as well and not just sit there and miss out. You’ll miss out on other things if you’re too busy with your mobile phone. I want to experience what is happening around me as well.*

Other participants emphasized choosing mindfulness and the importance of being mentally present, both in everyday life and in the digital sphere:

*Yes, I am a little bit different there. I try to do the opposite, I try to learn to be able to stand there and be bored for 5 min and not having to have to do something, and just be more*… *You know the whole mindfulness movement. I am kind of a little bit into that in a way. Thus, I try to not be addicted to constantly having something to entertain myself, or always have to be stimulated in a way, but just stand there and just*… *Yes*… *Be present in the environment and look around and just be*.

Participants described specific choices and actions they took to avoid being consumed by digital media, such as dinner or party member putting their phones into a “mobile bowl” until after the event. Another participant reflected:

*Yeah, like you mentioned the doctor’s office, when I’m at the doctor’s I often see people on their mobile phones, and I think to myself I’m not going to do that*… *(laughs). I don’t know, it sounds a bit like a know-it-all but, I just think that maybe this is an opportunity for me to sit and not do anything. It’s a bit relaxing to do that. I think I’m a bit influenced because I do yoga and I’m influenced by mindfulness, so I make choices like that and choose to rather spend my time on mindfulness than on my mobile.*

Thus, in contrast to the expressed tendency toward or worry about digital dependency, some participants expressed that they attempted a conscious effort toward digital moderation. They described deliberate strategies, such as voluntarily restricting mobile data access or engaging in moderate “digital detox” practices, to regain control over their attention and mitigate the adverse effects of constant connectivity. This represents a form of digital awareness, where users actively try to balance the benefits of technology with its overwhelming presence in their lives.

## Discussion

Here we provide answers to the two research questions by integrating the above insights with relevant research about the two sets of concepts, reflecting two main sets of tensions or paradoxes: *connectivity* (relationships and expectations) and *control* (dependence and awareness).

### RQ1: connectivity (relationships and expectations)

The participants experienced a fundamental and increasing paradox between the benefits of connectivity and the costs of social expectations to always be available and respond.

Since the launch of the iPhone in 2007 (Pierce, 2010), smartphones have evolved and converged into portable computing and multimedia hubs with easy internet access (see also Rice et al., 2023). Our findings reflect this shift. Participants expressed that they appreciated the capabilities and features of smartphones, particularly their ability to replace computers in many contexts. Nonetheless, in schoolwork contexts, the student participants expressed that computers were often deemed more functional and reliable. The theme of “context-dependent technology” revealed how these young adults relied on smartphones in daily life and on-the-go, benefiting from having essential tools in one device. The transformation of the mobile phone into a multifunctional smartphone—an example of *digital convergence*—appeared to have driven more intensive use, often at the expense of traditional computer use, and at the expense of more digital awareness and self-regulation.

From a positive perspective, digital media, especially mobile phones, enabled more continuous communication and heightened awareness of one’s social connections. The participants said that platforms made it easier for users to monitor or be notified about others’ activities. Hampton (2016, p. 118) noted that recent technologies offer “widespread affordances for persistent contact and pervasive awareness that have the potential to fundamentally change the structure of community.” As Jaff and Ciftci (2025, p. 2) summarize, “For many students, social media offers a platform for identity formation, self-expression, and the development of a supportive social network, all of which contribute to better mental health.”

However, their digital devices also increased responsibilities of and pressures for the individual to be available, maintain their relationships, and impose kinds of surveillance and control (Vanden Abeele et al., 2018; Table 1 above). The increased opportunities for connectivity came with the obligation to remain constantly available—a pressure they sometimes described as the “availability norm.” The student participants reported frustration with this persistent connectivity, and that the experienced pressure to be always available intruded on their private time. Some described social media as both engaging and exhausting, controllable yet overwhelming. They reported that their phones often caused more stress than pleasure—but they couldn’t part with them—and that their inability to keep up often generated anxiety. Others described that they felt “naked” without constant access.

Apparently, digital media capabilities and pervasive connectivity to one’s social and school networks reinforced FOMO, leading to increased anxiety and pressure to remain available (see also Fløtra, 2019). In turn, FOMO might drive persistent contact and awareness, further entrenching dependency. For example, Przybylski et al. (2013) found that individuals with high FOMO checked social media frequently—including during meals, lectures, and even while driving—while also experiencing ambivalence toward social media.

Social pressure to be available can reduce one’s self-control and sense of autonomy and competence (Halfmann and Rieger, 2019). Such expectations can increase perceived overdependence and guilt, reducing relational satisfaction (Hall and Baym, 2012). As Thulin and Vilhelmson (2007) concluded from their two-stage diary, interview, and panel study of 37 young adults in Sweden, “With the reduction in the constraints of time and space, the instant access of the mobile becomes difficult to refuse, and perceived dependency on mobiles increases” (p. 235). Studies by Cheever et al. (2014) and others have consistently linked frequent smartphone usage to elevated anxiety levels and other negative psychological outcomes. Many others have reported the negative impact of persistent contact and pervasive awareness on well-being (e.g., Hampton, 2016). Wang et al. (2025) noted student concerns about relationships between digital media use, privacy boundaries, and social expectations. Another study assessed interviews, observations, and a survey of Stanford University students, about their mobile phone practices. The results identified three themes: social expectation of constant connection, techno-social pecking order (decisions about interruptions and message responsiveness), and techno-resistance (trading off social censure with attempts to reduce constant connection) (Ames, 2013).

### RQ2: control (dependency and awareness)

The participants often questioned whether their digital media use was general usage, habit, dependency, or addiction. LaRose (2010) proposed viewing media habits on a continuum—from deliberate choices, to automatic responses to external stimuli. Habits are typically automatic, repeated behavior triggered by external cues. Rubin (2015) described habits as “the invisible architecture of daily life,” estimating that 40% of behavior is habitual. Applied to digital media use, this means both spontaneous interactions and more time-consuming activities may occur without conscious intent but also without dependency or addiction.

Concerns about mobile phone dependency were raised early on (e.g., Aoki and Downes, 2003), but the current ubiquity of the internet, smartphones, and social media has sparked increasing concern about their impact on adolescents and young adults, particularly regarding problematic usage (Shannon et al., 2022). Pellegrino et al.’s (2022) review of 501 publications between 2013 and 2022 documented how problematic or addictive social media use negatively affected young people’s mental health and wellbeing. Jaff and Ciftci (2025) also summarized research documenting relationships between dependency on social media platforms, reducing class attendance and study time, in some cases motivated by FOMO.

Media use becomes problematic when it interferes with other aspects of life, such as academic performance or general wellbeing—signs of dependency or even addiction. While public discourse often blurs the line between high media use and addiction, true media or internet addiction involves impaired control (Hagen and Wold, 2009). Young (1998) defined internet addiction as an impulse control disorder, with negative consequences for relationships, education, and employment. Gentile et al. (2013) argued that although there’s no formal diagnosis for technology addiction, the concept of pathological media use has robust construct validity. They emphasized the importance of distinguishing harmful use from healthy engagement. Beyond internet addiction, researchers have considered over-dependency on or addiction to social media. As an example, Chen et al. (2025), integrating survey and interview data, showed that Chinese college students’ social media anxiety increased general anxiety, which reduced one’s self-efficacy, while social media anxiety also fostered more negative and less positive coping strategies; over two-thirds reported “doomscrolling” and associated anxiety.

For most people, the terms “habitual use” and “dependency” are more applicable than clinical addiction. Nevertheless, the boundary is increasingly blurred as media companies craft ever more compelling apps and platforms (Weisberg, 2016). Social media platforms are intentionally designed to encourage habit formation and emotional rewards (Eyal and Hoover, 2014).

Despite these challenges, some participants described a sense of digital awareness. This theme of digital awareness coincides with emerging discussions in research literature centered on digital mindfulness and self-regulation. This mindset is gaining support in studies on digital detoxes and practices designed to foster healthier relationships with technology, such as digital backlash or the developing social movement to disconnect from digital media in various social spheres (see Albris et al., 2024; Syvertsen, 2020).

Some student participants manifested this awareness through mention of JOMO, the “joy of missing out,” increasingly recognized as an alternative to FOMO. JOMO was often described as associated with mindfulness, a practice of present-moment, non-judgmental awareness inspired by Jon Kabat-Zinn and rooted in Buddhist philosophy, and also yoga (see Hagen, 2018; Khoury et al., 2015; Pandey, 2023). This suggests applying more agency navigating media use. For example, Baym (2015) emphasized that while technology shapes behavior, individuals also possess significant agency in choosing how—and how much—to engage with it. Participants also expressed that they made conscious and reflected choices and resisted technological determinism (i.e., were better able to master media), actively shaping their digital lives; however, many more told that they succumbed to digital pressures (i.e., were more mastered by media).

The concept of *locus of control*, coined by Rotter (1954), offers another lens for understanding digital *media mastery*. It refers to an individual’s perceived agency. Those with an internal locus of control attribute outcomes to their own actions, while those with an external locus believe external forces are responsible. Internal control is generally associated with better health, resilience, and success (Ryon and Gleason, 2014; see also Tyler et al., 2020). There is also a positive relationship between internal locus of control and self-control (Botha and Dahmann, 2024). In Fløtra’s (2019) study, students who demonstrated internal control found it easier to regulate social media use while studying.

The discourse around digital media often seeks a balance—between work and leisure, digital life and physical presence. This longing is sometimes nostalgic, referring to a pre-digital “authentic” lifestyle (Syvertsen, 2020). Spending less time on digital media is viewed as linked to greater happiness, perhaps because it allows for more face-to-face interactions and better sleep (Twenge, 2019). While authorities and parents can attempt to monitor and manage children’s digital habits, young adults such as students are expected to self-regulate. Self-regulation—the capacity to override automatic thoughts or behaviors in favor of adaptive responses—is crucial for wellbeing (Gailliot et al., 2010; Hagen et al., 2018).

Several participants in our study cited mindfulness and yoga as ways of developing awareness and control of their digital media use. These disciplines emphasize self-reflection and personal responsibility for health and happiness (Hagen and Bhavanani, 2025). Jian et al. (2015) reported a mindful intervention, based in social cognitive career theory, in three Chinese universities about social media, digital literacy, and career competence. After a month of structured social media activities, the students reported greater self-regulation and the ability to perceive social media as a professional resource, not just as a source for social engagement and entertainment.

These perspectives do not absolve technology companies or regulators of responsibility—it simply underscores the value of *media mastery*: developing awareness, boundaries, and intentional strategies such as self-regulation to develop more mastery over, and reduce mastery by, digital devices that are providing affordances and tech companies providing premises for digital media uses, related experiences, and potential impact in students’, young adults’, and other people’s lives.

## Conclusion

### Main contribution

After providing an overview of the development of the media mastery framework, we have argued for its strengths as a sensitizing analytical lens. It incorporates a central paradox of digital media: the very tools designed to increase our mastery of connectivity, collaboration, and digital lives can also master us through dependence, social pressures, and fragmented attention. Complementing the inherent duality of the term “*media mastery*”—that is, the tension between being mastered by, and mastering, digital media—is the duality of the framework’s purpose. At a general conceptual level the media mastery framework represents a neutral descriptive framework (the bidirectional relationship between users and media), for example as applied to the focus group discussions below. However, it also represents a normative frame, whereby increased mastery of media, and reduced being mastered by media, should improve individual and social benefits of, while reducing harms from, digital media.

The key focus here is on understanding tensions and paradoxes in the processes of mastering, and being mastered by, digital media. Highlighting two of its central subcomponents, connectivity and control (and specific tensions within both), we show how applying the framework can illuminate several aspects of the digital lives of students. We have tried to illustrate student participants’ reported experiences where the benefits of digital convergence, such as communication and multifunctionality, were paradoxically accompanied by challenges, such as the pressure of constant connectivity and digital dependency. Thus, social connectivity can be a double-edged sword, involving both mastery and being mastered, by fostering richer, more accessible, and broader networks, while also heightening anxiety, expectations of perpetual availability, and dependency.

### Limitations

There are (at least) three limitations of this study. First, the results are based on just six focus group interviews, from one country. Digital media technology and social media use are context-dependent practices, thus encouraging deeper situational and even cross-cultural comparisons.

Second, these results reflect students’ thoughts and digital device experiences from a decade ago. To some extent, then, this study only provides an exploratory snapshot of the changing processes of university students’ experiences in being mastered by, and in attempting to master, their evolving digital devices. Researchers need to pay close attention to how digital media devices and platforms change over time, often with new affordances, constraints, and implied paradoxes for users (e.g., see Rice et al., 2023). Extending such a comparison across time, countries, and contexts could be a fertile ground for understanding how different social settings shape digital practices and perceptions, in a constantly changing media landscape.

Third, the full media mastery framework provides many more possible themes to pursue. Table 2 lists the most appropriate ones within the media mastery component.

**TABLE 2:** *Media mastery* sub-subcomponents also applicable to the focus group discussions.

Sub-component	Sub-subcomponents
Access	Access to medium/others/content, availability, convenience, notifications, low effort, social coordination ability
Boundaries	Balancing online and offline self and networks, blending/blurring, constant connection, continuous co-presence, identity disjuncture, perpetual and persistent contact, privacy, responsibility, safety, ubiquity, vulnerability, work/non-work
Constraints	Contradiction/paradox/tension, loss or change of some traditional skills or relations, unintended consequences
Managing content	Awareness, consumption, control over own content, gratifying, media literacy, media multitasking, personal information, producers, temporary or ephemeral
Obstacles	Physical access, battery, break/lose, updating, compatibility, complexity, connections, costs, distracting, frustration, overload, interference, interruptions, tech problems, techno-stress, viruses/malware
Use awareness	Attitudes about one’s use, balance of active or passive use, balancing self and group, choices about how and when to use, media comparisons, media convergence, media habit, meta-attention, monitoring or checking frequently, preparing responses, self-regulation, strategizing media use for coordination, taken-for-grantedness, techno-resistance, tool awareness, use of multiple media

As discussed, the media mastery framework includes four components of occasions or factors for *media mastery*, and a media mastery component. Many aspects of these five components could be identified in the focus groups, though this paper considers only a few: relationships (connectivity and expectations) and control (dependence and addiction). Within the media mastery component, the above subcomponents and their sub-subcomponents could also possibly be applied to the focus group discussions.

### Implications

Insights from 2015 to 2016 focus group interviews in Norway indicated the primary role of digital devices like PCs and mobile phones in the lives of young adults, represented here through BA level university students. During the last decade, these devices, especially smart phones, have become more integrated in daily life, as they are used for managing almost everything: connecting, checking time, checking social messages, booking, paying, taking the bus, parking, weather, photographs and videos, music, etc. Even a decade ago, the interviewees appreciated the potential for connectivity, but also experienced its disadvantages, like availability norms and constant interruptions. The 2015–2016 data also pointed to the increased need for control of digital devices, as increasing awareness is challenging the dependency and addiction users may develop toward their digital devices, especially smart phones.

There is a continued need for policy development, theoretical frameworks, practical designs, and individual guidelines that can help individuals navigate the paradoxes and tensions inherent in digital media capabilities and uses. As society is increasing the digitization—recently with a focus on AI—across all sectors, researchers, teachers, technology companies, and policymakers need to consider and reflect on how society makes it nearly impossible for its members to live without digital devices. This includes understanding how technology companies, advertisers, and others try to influence and manage users, using strategies to attract and keep users engaged, as they have obvious and not obvious commercial and governmental interests in maintaining relations with users in order to sell user information to advertisers, service designers, and law enforcement agencies (Zuboff, 2019). There is a need to regulate the role that digital media platforms can play in people’s lives, a process that has started related to banning or limiting uses of social media by children under varying ages (e.g., Australia, France, Germany, Greece, Norway, Slovenia, Spain, and several U.S. States; see Reuters, 2026).

At the same time, an important question is what role users and their fellow users, and researchers, can play in fostering greater awareness and resistance to the persuasive strategies employed by digital platforms and commercial companies (AbedRabbo et al., 2025). The emotional and psychological reliance on these devices prompts further questions about balance and agency. Concepts such as *mindfulness*, *locus of control*, and *self-regulation* may serve as valuable strategies, enriching the understanding of *media mastery*. For instance, how might digital literacy training or mindfulness interventions (Twenge, 2019) help mitigate these dependency effects? Yoga was occasionally mentioned as a mean to cope with dependency and addiction. Indeed, “By encouraging self-control, yoga is regarded as one of the effective methods for paving the road for digital detoxification from technology and smartphone addiction” (Jagadambal, 2025, p. 1). Educators, policymakers, researchers, and users may need to explore how mindfulness and yoga can play a role in fostering *media mastery* and creating healthier digital ecosystems, by facilitating balance, and reducing current health challenges and inherent paradoxes of digital device usage.^2^

## Data Availability

The raw data (focus group transcripts) supporting the conclusions of this article are available from the first author upon request, without undue reservation.

## Footnotes

1 The 2015/2016 Norwegian focus groups study received approval by the Norwegian Social Science and Data Services (project #45345). Participants were provided with and signed informed consent forms. The focus groups also served as the foundation for the theses by Aanby (2017), Fløtra (2019). The first author moderated the interviews, with help from student assistant Esi Hewton, who also recruited the participants. Hewton made significant contributions to the *media mastery* project, including the analysis and a summary report of the 2015/2016 focus group interviews.

2 This study received no Grant funding but did get Department support for the student assistant. The authors report there are no competing interests to declare. Focus group transcripts are available from the corresponding author upon reasonable request.
